# The Low-Cycle Fatigue Behavior of a High-Strength Low-Alloy Steel Subjected to Tempforming

**DOI:** 10.3390/ma18050972

**Published:** 2025-02-21

**Authors:** Anastasiia Dolzhenko, Pavel Dolzhenko, Valeriy Dudko, Rustam Kaibyshev, Andrey Belyakov

**Affiliations:** Laboratory of Mechanical Properties of Nanostructured Materials and Superalloys, Belgorod State University, Belgorod 308015, Russia; dolzhenko_a@bsuedu.ru (A.D.); dolzhenko_p@bsuedu.ru (P.D.); dudko@bsuedu.ru (V.D.); rustam_kaibyshev@bsuedu.ru (R.K.)

**Keywords:** high-strength low-alloy steel, tempforming, low-cycle fatigue, strain softening

## Abstract

The developed microstructures and their deformation behavior were studied in a high-strength low-alloy steel subjected to tempforming, i.e., tempering followed by large-strain rolling at temperatures of 823 K or 923 K. Tempforming has been recently proposed as an advanced treatment for low-alloy steels in order to substantially increase their impact toughness at low temperatures. However, the mechanical properties, especially the fatigue behavior, of tempformed steels have not been studied in sufficient detail. The present study, therefore, is focused on the strengthening mechanisms of the tempformed steel, placing particular emphasis on the low-cycle fatigue behavior. Tempforming resulted in a lamellar-type microstructure with a high dislocation density and dispersed Cr_23_C_6_ carbide particles. The size of the latter particles increased from 25 nm to 40 nm with an increase in tempforming temperature. The transverse grain size and dislocation density comprised 550 nm and 2.6 × 10^15^ m^−2^ after tempforming at 823 K or 865 nm and 1.8 × 10^15^ m^−2^ after processing at 923 K, respectively. Tempforming led to significant strengthening, which was attributed to high-density dislocations arranged in low-angle subboundaries. The yield strength of 1140 MPa or 810 MPa was observed for the steel samples tempformed at 823 K or 923 K, respectively. The low-cycle fatigue behavior depended on the plastic strain amplitude, which, in turn, was controlled by the previous strengthening under tempforming conditions besides the total strain amplitude. An increase in the plastic strain amplitude promoted fatigue softening that was caused by a decrease in the dislocation density as a result of subgrain coalescence.

## 1. Introduction

The treatment of tempforming, consisting of tempering followed by large-strain warm rolling, has been recently proposed to improve the impact toughness of high-strength low-alloy steels at low temperatures [1,2]. Tempformed steel samples are characterized by lamellar-type microstructures composed of highly elongated grains with a transverse size of hundreds of nanometers, with finely dispersed oxides and high dislocation densities [3,4]. The mechanisms of microstructure evolution in low-alloy steels during large-strain warm deformation have been fairly clarified [5,6,7]. The tempformed microstructures result from a kind of continuous dynamic recrystallization, namely, the development of strain-induced subboundaries and the progressive increase in their misorientations up to the typical values of high-angle grain boundaries, as well as the arrangement of the grain boundaries along the rolling plane after sufficiently large rolling reductions [6,8,9,10,11,12]. Note here that the microstructure evolution during warm deformation can be remarkably accelerated by using an initial martensitic microstructure [13]. Tempforming at lower temperature results in finer grains with smaller dispersed particles and higher dislocation density [3,14]. Tempformed steel samples exhibit an outstanding impact toughness especially at low temperatures that has been attributed to the delamination phenomenon resulting in notch blunting, owing to easy splitting crosswise to the principal crack propagation [15,16]. The mechanical performance of the tempformed steels is governed by the developed microstructure under processing conditions. Besides the ultrafine-grained microstructure, warm rolling with large total reductions results in a strong fiber texture with <110> parallel to the rolling direction (RD) and <100> crosswise to the RD that is typical of the unidirectional rolling of bcc metals/alloys [17]. Hence, cleavage fractures readily occur on the longitudinal {100} planes of the tempformed steel samples, leading to delamination during impact tests at lowered temperatures [18]. Such crack branching along the impact test specimen substantially increases the absorbed energy and corresponding impact toughness.

One of the important practical applications of tempforming is the principal possibility of improving the mechanical performance of low-alloy and carbon steels with high impurity. In spite of their attractively low price, such steels suffer from brittle fracture because of grain boundary segregations, mainly of phosphorus and sulfur, and the consequent loss of boundary cohesion [19,20]. On the other hand, easy splitting along the boundaries crosswise to the direction of the main crack propagation may promote the delamination toughness of steels subjected to tempforming. Jafari et al. [21,22] and Min et al. [23] successfully utilized the detrimental effect of phosphorus segregation to enhance the impact toughness of phosphorus-doped steels by means of tempforming treatment. The phosphorus segregations at grain boundaries and {100} planes arranged perpendicular to the notch orientation effectively accelerated delamination and enhanced impact toughness in phosphorus-doped steels with ultrafine elongated grain structures after tempforming.

In addition to impact toughness, tempforming significantly increases the strength of low-alloy steels [3,4]. Depending on alloying extent (mainly carbon content), tempforming provides an increment of the yield strength by 20% to 60% [2,4,14,24]. Note that low-carbon steels are more susceptible to tempforming strengthening. The microstructural mechanisms responsible for the strengthening in the course of tempforming have been a subject of several recently published research papers [2,3,4,7,16,24,25,26,27,28]. Commonly, several special strengthening mechanisms are considered to operate in tempformed steels. Those are grain refinement, work hardening and dispersed strengthening. The contribution of the individual mechanisms to the overall strength depends on the steel chemical/phase content and the processing conditions [3,4,16,28]. On the other hand, the different mechanisms were shown to correlate with each other from the viewpoint of their strengthening effect [4]. For instance, decreasing the tempforming temperature increases the strength, owing to all the strengthening contributors. A unique beneficial combination of mechanical properties including high strength and impact toughness, which, surprisingly, tend to increase with a decrease in temperature, makes tempforming a very promising method for the production of structural steels for various reliable constructions/fasteners serving at low ambient temperatures, e.g., in the polar region [2,29].

It should be noted, however, that the mechanical properties of tempformed steels have not been studied comprehensively. Tempforming is a relatively new, just proposed method, and most of the current studies deal with the impact and tensile tests of tempformed steels. On the other hand, labor-intensive and time-consuming mechanical tests, like those for the creep and fatigue behavior, have not been investigated for steels subjected to tempforming. Therefore, the aim of the present work is to clarify the mechanical behavior of a high-strength low-alloy steel subjected to tempforming, focusing on the microstructural response of tempformed steel with ultrafine elongated grains involving a high dislocation density and finely dispersed oxides in the cyclic loading. The paper places particular emphasis on the effect of different mechanisms on strength and its degradation under low-cycle fatigue conditions. The effects of tempforming strengthening and cyclic strain amplitude on mechanical performance are considered in some detail.

## 2. Materials and Methods

A steel with the chemical composition of 0.15% C, 1.32% Mn, 1.42% Cr, 0.45% Mo, 0.42% Cu, 0.17% Ti, 0.007% S and 0.008% P (all in wt%), produced by “SMSM”, Ltd., Moscow, Russia, was heated to 1123 K, water quenched and tempered for 1 h at 823 K or 923 K, followed by multiple rolling with a thickness reduction from 45 mm to 10 mm (about a 20% reduction in each rolling pass with a strain rate of about 1 s^−1^) at the tempering temperature to a total strain of 1.5, followed by air colling. The selected tempering temperatures were in the range of appropriate temperatures for the tempforming of high-strength low-alloy steels as suggested in previous studies [1,2,3,4,16]. The specimens for the tensile and fatigue tests were machined along the RD. Three tensile specimens with a gauge length of 12 mm and a cross section of 3.0 mm × 1.5 mm were tensioned with a crosshead rate of 2 mm/min using an Instron 5882 testing machine for each data point. The specimens with a gauge length of 8 mm and ∅5 mm were subjected to fatigue tests. The low-cycle fatigue tests were carried out with symmetric tension–compression loading (R = −1) under the strain control mode with strain amplitudes, Δε/2, of 0.6% and 0.85% (one specimen for one data point) and a frequency of 2 Hz, using an Instron 8801 testing machine.

The microstructural observations were carried out on the longitudinal sections using a Quanta 600 FEG scanning electron microscope (Quanta, Houston, TX, USA) equipped with an electron backscattered diffraction (EBSD) pattern analyzer (Oxford Instruments, High Wycombe, UK) incorporating an orientation imaging microscopy (OIM) with TSL OIM software (ver. 6.2) and a JEOL JEM-2100 transmission electron microscope (TEM) (JEOL, Tokyo, Japan). The OIM images were subjected to a cleanup procedure using the Grain Dilation method, setting a grain tolerance angle of 2° and a minimal grain size of 12. The minimal area covered by the OIM map was 2500 μm^2^ per each steel sample, with a mapping step size of 50 nm. The transverse grain size was evaluated on the OIM images as the average distance between high-angle grain boundaries with misorientations of θ ≥ 15°. The local misorientations in the substructures were measured by the Kikuchi-line method, using the converged beam TEM technique [30]. The phase content at different temperatures was calculated by Thermo-Calc software using the TCFE-7 database.

## 3. Results

### 3.1. Tempformed Microstructures

**Figure 1:**
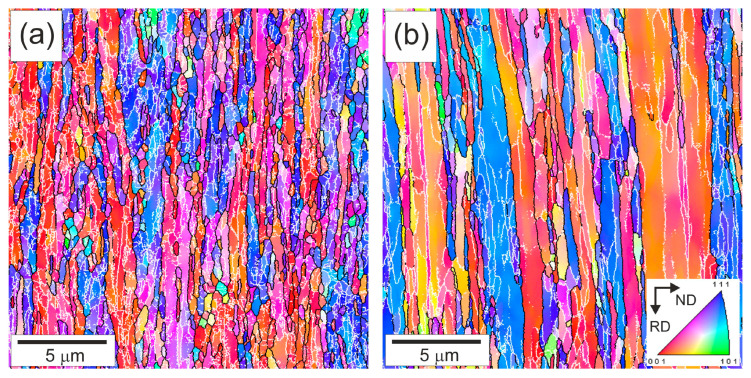
OIM images of microstructures after tempforming at 823 K (**a**) or 923 K (**b**). High-angle grain boundaries (θ ≥ 15°) and low-angle subboundaries (2° ≤ θ < 15°) are indicated by black and white lines, respectively. Colors indicate directions along normal direction (ND).

The typical microstructures developed in the steel samples by tempforming at 823 K or 923 K are shown in Figure 1. The mechanism of microstructure evolution during tempforming has been considered as a kind of continuous dynamic recrystallization in many previous studies [3,4,5,6,7]. The main regularities, like temperature and strain dependencies, have been fairly established [11,12]. Let us consider the developed microstructures in the present steel from the viewpoint of the operating strengthening mechanisms. The developed microstructures consist of highly elongated grains towards the RD that are a common feature of large-strain plate rolling under the conditions of cold to warm deformation. The OIM images in Figure 1 show orientation distribution along the normal direction (ND). Two fiber textures of <111>//ND and <001>//ND are clearly seen in Figure 1 for both temperatures. Similar microstructures were frequently observed in other steels subjected to large-strain warm rolling [3,4,6,13]. The difference in the microstructures evolved during tempforming at 823 K or 923 K is associated with the transverse grain/subgrain size. Processing at lower temperature results in smaller grain/subgrain sizes. The transverse grain size is 550 nm after tempforming at 823 K, whereas that after tempforming at 923 K is 865 nm (Figure 1). The smaller subgrain size evolved at lower tempforming temperature results in a larger subboundary area per unit volume (S_V_). The latter was considered as the main site for dislocation storage in the case of well-developed dislocation substructures [31,32,33]. The corresponding dislocation density can be evaluated as ρ_LAB_ = 1.5 S_V_ θ_LAB_/b, where θ_LAB_ is the average misorientation of the low-angle dislocation subboundaries and b is the Burgers vector [32]. The present values of S_V_ and θ_LAB_ in the steel samples tempformed at 823 K comprise 4.8 × 10^6^ m^−1^ and 5.2°, respectively; those after tempforming at 923 K are 2.9 × 10^6^ m^−1^ and 5.9°. Then, the dislocation densities of ρ_LAB_ = 2.6 × 10^15^ m^−2^ and ρ_LAB_ = 1.8 × 10^15^ m^−2^ could be calculated in the samples after tempforming at 823 K or 923 K. Thus, the dislocation density in the steel sample tempformed at 823 K is almost 1.5 times higher than that in the sample tempformed at 923 K, providing an increment in dislocation strengthening of about 20%. It is worth noting that a lower dislocation density corresponds to larger grain sizes, much like in other studies on the thermo-mechanical treatment of steels and alloys [6,34,35].

**Figure 2:**
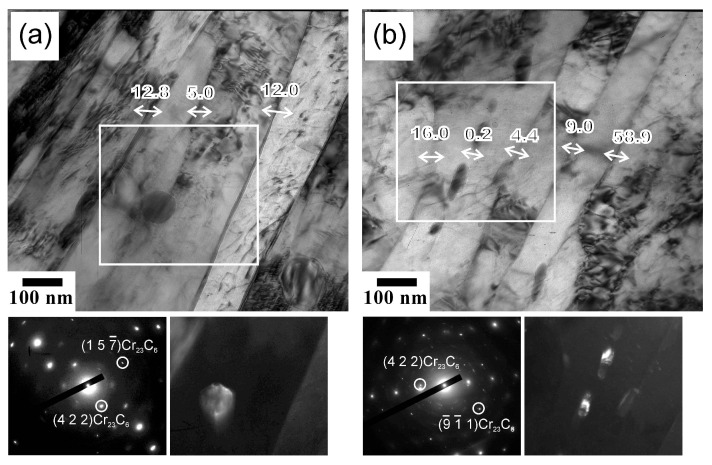
TEM images of the fine substructures evolved during tempforming at 823 K (**a**) or 923 K (**b**). The numbers indicate the grain/subgrain boundary misorientations in degrees. The selected portions are corresponded with dark-field images of the Cr_23_C_6_ carbide particles. The diffraction patterns were obtained from selected areas of ∅125 nm.

The fine substructures evolved during tempforming at different temperatures are shown in Figure 2. The microband-like grains/subgrains in Figure 2 are bounded by low-to-high angle (sub)boundaries. It is worth noting that the low-angle subboundaries are characterized by a wide range of misorientations up to 15°, corresponding well to the relatively large average subboundary misorientations of about 5° revealed by OIM in Figure 1. Numerous dispersed Cr_23_C_6_ particles are observed in the processed steel samples, as clearly seen in Figure 2. The particles are located at various grain/subgrain boundaries irrespective of the (sub)boundary misorientation, including at the dislocation subboundaries with quite small misorientations. The average size of these particles increases from about 25 nm to 40 nm as tempforming temperature increases from 823 K to 923 K. Almost the same carbide particle sizes were reported in other studies on tempforming at 823–923 K [3,36]. The volume fractions of Cr_23_C_6_ at 823 K and 923 K are 0.018 and 0.016, respectively, as calculated by Thermo-Calc (ver. 5). Besides Cr_23_C_6_, Thermo-Calc predicts a small amount of TiC, about 0.0025. Therefore, the total volume fraction of the dispersed particles in the present steel samples can be taken as 0.02 at 823 K and 0.019 at 923 K.

### 3.2. Mechanical Tests

**Figure 3:**
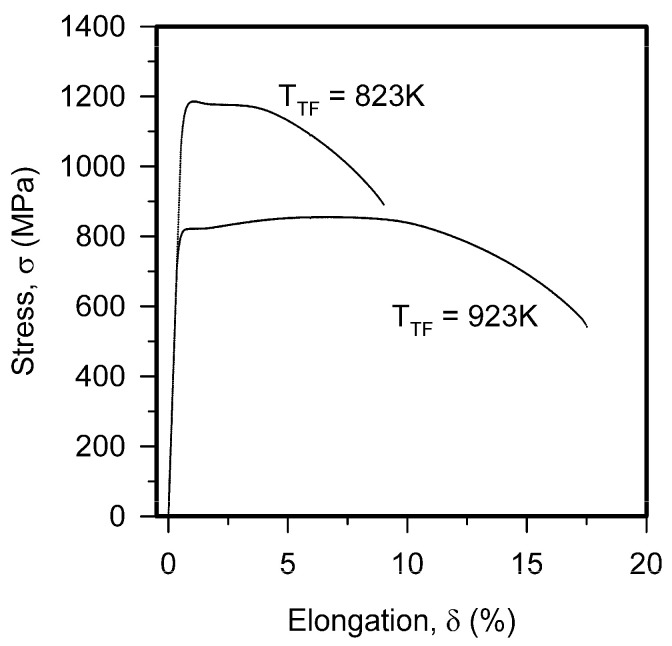
Engineering stress—elongation curves obtained during tensile tests of high-strength low-alloy steel subjected to tempforming at indicated temperatures (T_TF_).

The engineering stress vs. elongation curves for the specimens made of the steel tempformed at 823 K or 923 K are shown in Figure 3. A high yield strength (σ_0.2_) of 1140 MPa and ultimate tensile strength (UTS) of 1190 PMa are obtained after tempforming at 823 K. Following the yielding, the tensile stress shortly increases to its maximum, followed by a small steady-state to about 3% elongation, and then gradually decreases (necking) up to failure at about 8% elongation. On the other hand, a relatively low yield strength of 810 MPa and a remarkable strain hardening stage (uniform elongation of around 8%) leading to UTS = 850 MPa are observed after tempforming at 923 K. Hence, a decrease in tempforming temperature decreases the strength but promotes plasticity. The total elongation of 17% after tempforming at 923 K is almost double of that after processing at 823 K.

**Figure 4:**
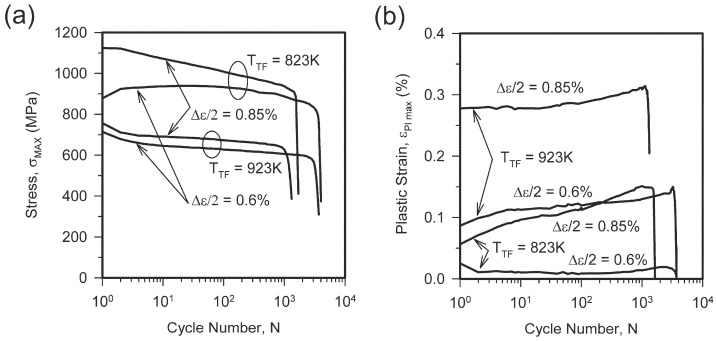
Fatigue dependencies of maximal stress (**a**) and maximal plastic strain (**b**) based on cycle number for high-strength low-alloy steel samples subjected to tempforming at temperatures of T_TF_ = 823 K or T_TF_ = 923 K.

The changes in the maximal flow stress (σ_MAX_) and the plastic strain (ε_Pl max_) during fatigue tests with strain amplitudes (Δε/2) of 0.6% and 0.85% are shown in Figure 4. It is worth noting that specimens made of steels subjected to tempforming at lower temperature are characterized by higher strengthening levels and withstand a larger number of fatigue cycles (N) under the same strain amplitude. The maximal stress at the first cycle (N = 1) with a relatively large strain amplitude of 0.85% is close to the corresponding yield strength. On the other hand, σ_MAX_ at N = 1 decreases with a decrease in strain amplitude, especially for the sample which was significantly strengthened at a low tempforming temperature of 823 K. The σ_MAX_ vs. N curves exhibit two types of behavior (Figure 4a). Namely, gradual softening is observed through the fatigue tests for the samples tempformed at 923 K irrespective of Δε/2 and those tempformed at 823 K and tested with Δε/2 = 0.85%, while a small softening occurs after about one hundred cycles with Δε/2 = 0.85%, following almost constant σ_MAX_ in the early fatigue stage. It is clearly seen in Figure 4b that the change in the plastic strain correlates with that of the maximal flow stress. Namely, an increase in the plastic strain during the fatigue tests is accompanied by a decrease in the maximal flow stress. Therefore, the fatigue softening is controlled by the plastic strain amplitude (ε_Pl max_). The softening occurs during fatigue tests under conditions of rather large plastic strain, above about 0.1%. In contrast, σ_MAX_ hardly changes during the tests, with small plastic strains well below 0.05%.

### 3.3. Fatigue Microstructures

**Figure 5:**
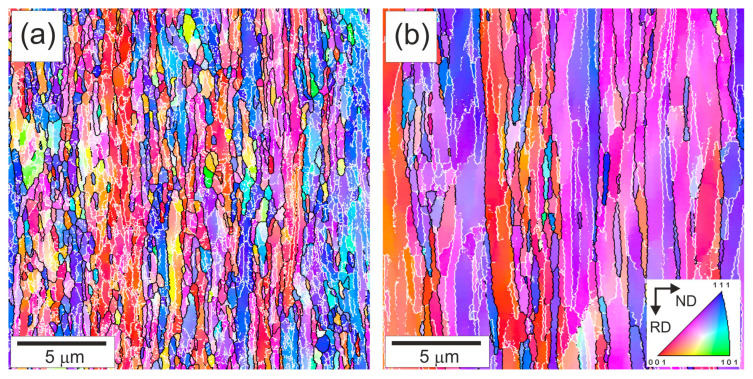
OIM images of the microstructures after tempforming at 823 K (**a**) or 923 K (**b**) and then subjected to fatigue tests with a strain amplitude of 0.6%. High-angle grain boundaries (θ ≥ 15°) and low-angle subboundaries (2° ≤ θ < 15°) are indicated by the black and white lines, respectively. The colors indicate directions along the normal direction (ND).

The representative microstructures of the specimens after the fatigue tests are shown in Figure 5 as OIM images. The original rather strong two-component texture of <111>//ND and <001>//ND that developed by large-strain warm rolling hardly changed during the fatigue tests. The lamellar-type microstructures after the fatigue tests are qualitatively very similar to those evolved by tempforming, although the fatigue conditions affected the change in microstructural parameters. The transverse grain size (550 nm) after tempforming at 823 K and that of 569 nm after a subsequent fatigue test with a small Δε/2 of 0.6% (Figure 5a) are almost the same. In contrast, the transverse grain size increases to 1.08 μm during the fatigue test following tempforming at 923 K (Figure 5b). The specimens after the fatigue tests are characterized by a relatively high dislocation density, which is mostly associated with numerous low-angle dislocation subboundaries (Figure 5). The corresponding dislocation densities comprise ρ_LAB_ = 2.5 × 10^15^ m^−2^ and ρ_LAB_ = 1.5 × 10^15^ m^−2^ in the fatigued specimens tempformed at 823 K or 923 K, respectively. The change in the dislocation density during the fatigue tests is caused by a decrease in S_V_ to 4.6 × 10^6^ m^−1^ and 2.4 × 10^6^ m^−1^ while the θ_LAB_ slightly increases to 5.3° and 6.2° in the samples tempformed at 823 K or 923 K, respectively (Figure 5). Therefore, the change in microstructural parameters during the fatigue testing of tempformed steel depends remarkably on the tempforming conditions. Processing at a relatively low tempforming temperature increases the strength and promotes microstructural stability upon subsequent fatigue tests.

**Figure 6:**
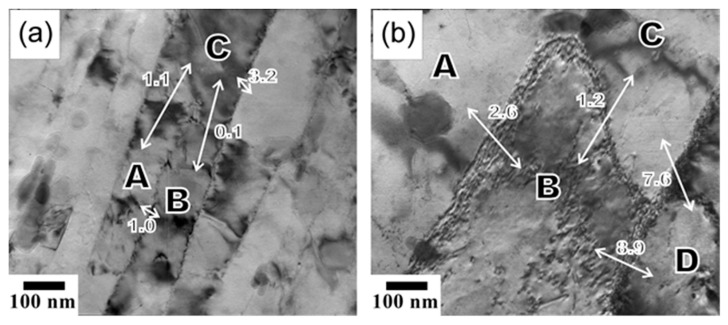
TEM images of the dislocation substructures in fatigued specimens (Δε/2 = 0.6%) made of steel samples tempformed at 823 K (**a**) or 923 K (**b**). The numbers indicate the misorientations in degrees between the pointed (lettered) regions.

The fine substructures in the specimens made from steel samples tempformed at 823 K or 923 K and then fatigue tested with a small Δε/2 = 0.6% are shown in Figure 6. The dislocation substructures are represented by highly elongated subgrains, as originated from large-strain warm rolling. It should be noted that the ultrafine elongated grains/subgrains in Figure 6 are characterized by a quite small number of dislocations in their interiors. Most of the dislocations arrange along the dislocation subboundaries. This is very similar to the dislocation substructures after recovery annealing [33,37]. That is, dynamic recovery during cyclic loading resulted in the dislocation rearrangement. Let us consider the evolution of the dislocation substructure during fatigue testing in more detail. The dislocation subboundary with a misorientation of θ = 1° between the regions labeled A and B terminates toward region C (Figure 6a). This subboundary misorientation of 1° is compensated by elastic distortion, as suggested by bend contours between A and C, whereas such distortion between B and C is negligibly small. Therefore, the disintegration of this subboundary between A and B due to moving the constituting dislocations across A towards the opposite subboundary should decrease the total dislocation density and promote the release of internal stresses in the specimen. Such dynamic subgrain coalescence is more pronounced in the fatigued (Δε/2 = 0.6%) specimen made from the steel sample tempformed at 923 K (Figure 6b). The motion of the dislocation subboundary between B and C with θ = 1.2° in Figure 6b towards B results in a remarkable decrease in the dislocation density, owing to removing the dislocation subboundary (θ = 2.6°) between A and B and decreasing the misorientation between B and D from 8.9° to 7.6°. Hence, the dislocation redistribution powered by cyclic straining leads to dynamic subgrain coalescence, which can be considered as the main mechanism responsible for fatigue softening in tempformed steels.

## 4. Discussion

The present steel samples subjected to tempforming exhibit high strength in the range of 800 MPa to 1200 MPa depending on their processing temperature. Generally, the strengthening of high-strength low-alloy steels is attributed to several mechanisms [38]. Among those, the following should be considered in the present case: solid solution strengthening, grain size (grain boundary) strengthening, dispersion (Orowan) strengthening and dislocation strengthening (work hardening). Solid solution strengthening (σ_SS_), depending on alloying extent, may provide a remarkable increment for the yield strength, which can be calculated using the following empirical equation [38,39].

σ_SS_ = 32Mn + 678P + 83Si + 39Cu − 31Cr + 11Mo + 5544C, (1)

The numbers here correspond to the weight percentage. The solid solution in steels depends significantly on the second-phase precipitations at their tempering temperature. According to Thermo-Calc, the solid solution depletion of the present steels at the tempforming temperatures is conditioned by the decreasing of 10^−2^–10^−4^C, 0.7–0.9Cr, 0.1–0.2Mo, 0.1–0.4Cu and 10^−6^Ti (note that the smaller concentrations indicated in wt% correspond to lower temperatures, and vice versa). Thus, the solid solution strengthening of the present steels after tempforming at 823 K or 923 K was 70 MPa or 81.5 MPa, respectively. Grain size strengthening (σ_G_) is commonly related to the second term of the Hall–Petch equation [40,41,42].

σ_G_ = k D^−0.5^ (2)

Here, k of 0.24 MPa m^0.5^ is the grain boundary strengthening factor for low-carbon steels [43]. A corresponding strengthening of σ_G_ = 324 MPa or σ_G_ = 258 MPa was obtained for the present steel samples subjected to tempforming at 823 K or 923 K, respectively. Dispersion strengthening (σ_Or_) can be evaluated using the Orowan mechanism [44].

σ_Or_ = 0.2 MGb λ^−1^ ln(d*/b), (3)

where M of 2.75 is the Taylor factor for the bcc lattice, G is the shear modulus, λ is the particle spacing (edge-to-edge) and d* depends on λ and particle size (d_p_) as d* = (d_p_^−1^ + λ^−1^)^−1^. The dispersion strengthening for the present steels comprises σ_Or_ = 488 MPa and σ_Or_ = 326 MPa after tempforming at 823 K or 923 K, respectively. Dislocation strengthening (σ_ρ_) is usually expressed by the Taylor-type equation as a square root function of the dislocation density (ρ) [45,46,47].

σ_ρ_ = αGb ρ^0.5^, (4)

where α is a numerical factor of about 0.9 for low-carbon steels [48,49]. Taking the dislocation density associated with numerous low-angle subboundaries, i.e., ρ_LAB_, dislocation strengthening values of 929 MPa and 773 MPa can be obtained for the steel samples tempformed at 823 K or 923 K, respectively. It is worth noting that the obtained dislocation strengthening values are almost the same as the yield strength (without the solid solution and the lattice friction of about 40 MPa [38]), and it is comparable with a summation of other strength contributors, namely, the grain size strengthening (324 MPa for 823 K and 258 for 923 K) and dispersion strengthening (488 MPa for 823 K and 326 MPa for 923 K). The small difference may be associated with some overestimation of dispersed particle size because the smaller particles are easier to miss. This is in line with an approach recently presented by Takaki et al. for microstructural strengthening [49]. Namely, the flow stresses are solely conditioned by dislocation motion, which in turn depends on all other retarding factors like dispersion strengthening, etc. The overcoming of the latter factors should determine the overall dislocation density. This makes various strengthening mechanisms interdependent. Another frequently used approach to strengthening assumes that the microstructural strength contributors are independent and linearly additive [32,38]. However, in this case, matching with experimental results may lead to significantly reduced values of strengthening coefficients, i.e., k in Equation (2) and/or α in Equation (4) [50,51].

The dislocation density in the tempformed steel samples decreases during fatigue tests. Correspondingly, the dislocation strengthening (Equation (4)) decreases to 911 MPa and 706 MPa in steels tempformed at 823 K or 923 K. The steel sample tempformed at 823 K is characterized by a small decrease in dislocation strengthening that corresponds to a quite small softening on the σ_MAX_–N fatigue curve in Figure 4a. In contrast, following tempforming at 923 K, a remarkable decrease in dislocation density during fatigue testing reduces the corresponding dislocation strengthening by about 70 MPa that roughly matches the fatigue softening of about 100 MPa in Figure 4a. A decrease in the dislocation density during the fatigue tests is accompanied by a small increase in the transverse grain size. Correspondingly, σ_G_ (Equation (2)) decreases to 318 MPa and 231 MPa in steels tempformed at 823 K or 923 K. Again, the change in strength during fatigue tests can be attributed to the change in dislocation density. The remarkable decrease in the subboundary area per unit volume (S_V_) along with some increase in the average subboundary misorientation (θ_LAB_) during fatigue tests testify to a kind of dynamic subgrain coalescence as the microstructural mechanism responsible for the fatigue behavior of the present steels after tempforming.

The present results of the mechanical tests suggest that tempforming is indeed a unique treatment providing inclusive enhancement of the mechanical properties of low-alloy high-strength steels. Besides increasing the impact toughness, as reported in previous studies [2,3,4,5,14,15,16,26,27,28,29], an increase in the tensile strength by tempforming is accompanied by improving the low-cycle fatigue resistance. Note that improving the fatigue behavior of high-strength low-alloy steels after tempforming should be further confirmed by stress-controlled high-cycle tests as well as fracture toughness tests. On the basis of the current results, however, there is no doubt that tempforming can be part of an advanced technological approach to producing inexpensive low-alloy steels with enhanced mechanical properties designed for low-temperature constructions.

## 5. Conclusions

The deformation behavior of a high-strength low-alloy steel subjected to tempforming at 823 K or 923 K was studied. The main results can be summarized as follows.

1. Tempforming resulted in the development of highly flattened grains with a high dislocation density in numerous low-angle subboundaries and dispersed carbides. The transverse grain size increased from 550 nm to 865 nm with an increase in tempforming temperature from 823 K to 923 K. The corresponding dislocation density decreased from 2.6 × 10^15^ m^−2^ to 1.8 × 10^15^ m^−2^, and the average carbide particle size increased from 25 nm to 40 nm.
2. The development of an ultrafine-grained lamellar-type microstructure with a high dislocation density and oxide dispersion during tempforming at 823 K or 923 K provided yield strengths of 1140 MPa and 810 MPa, respectively. The dislocation density that evolved during tempforming resulted in strengthening comparable with the yield strength and with the strengthening by the grain size and dispersed particles.
3. The change in the maximal stress during low-cycle fatigue tests depended on the maximal plastic strain, which increased with a decrease in the strengthening by previous tempforming. An increase in the plastic strain stimulated the fatigue softening. Low-cycle fatigue tests with the plastic strain above 0.05% were accompanied by remarkable softening, whereas almost the same maximal stresses were observed until several hundred cycles with smaller plastic strain.
4. The fatigue softening was attributed to the decreasing of the dislocation density as a result of dynamic subgrain coalescence, which was promoted by fatigue plastic strain.

## Data Availability

Data is part of the ongoing research and will be made available on request.
